# Unusually high incidence of polyomavirus JC infection in the higher grade of colorectal cancer tissues in Taiwan

**DOI:** 10.1186/s40001-022-00756-2

**Published:** 2022-07-20

**Authors:** Chuan-Yin Fang, San-Yuan Chen, Bo-Xiu Hsiao, Hsin-Yi Huang, Yi-Ju Chen, Chun-Liang Tung, Chiung-Yao Fang

**Affiliations:** 1 Division of Colon and Rectal Surgery, Ditmanson Medical Foundation Chia-Yi Christian Hospital, Chiayi, 621 Taiwan; 2 Department of Chinese Medicine, Ditmanson Medical Foundation Chiayi Christian Hospital, Chiayi, Taiwan; 3 Department of Sports Management, Chia Nan University of Pharmacy & Science, Tainan City, Taiwan; 4 Department of Medical Research, Ditmanson Medical Foundation Chiayi Christian Hospital, 539, Chung Hsiao Road, Chiayi, 600 Taiwan; 5 Department of Pathology, Ditmanson Medical Foundation Chiayi Christian Hospital, Chiayi, Taiwan; 6 Department of Food Nutrition and Health Biotechnology, Asian University, Taichung, 413 Taiwan

**Keywords:** JCPyV, Colorectal cancer, Nested PCR, Tumor progression, DNA virus

## Abstract

**Introduction:**

The human JC polyomavirus (JCPyV) has been detected in colorectal cancer (CRC) tissues and is suggested to contribute to CRC tumorigenesis. The rearrangement of the JCPyV regulatory region is supposedly associated with CRC development. The progression of CRC involves the stepwise accumulation of mutations. The large tumor antigen (LT) of JCPyV can trigger uncontrolled cell cycle progression by targeting oncogenes, and tumor suppressor genes, and causing chromosome instability. Few studies have focused on the presence of JCPyV DNA in the higher grade of CRC tissues.

**Methods:**

We collected 95 tissue blocks from samples of stages I, II, III, and IV CRC. Nested PCR targeting the regulatory region of the viral genome was performed to determine the presence of JCPyV DNA in the various stages of colorectal cancer tissues.

**Results:**

The nested PCR results showed that the positive rate of JCPyV DNA increased with the progression of CRC stages. The archetypal-like, non-rearrangement genotype of JCPyV with subtle mutations was the major genotype found in CRC samples.

**Conclusions:**

This finding in our study suggests that there may be an association between JCPyV and CRC progression.

**Supplementary Information:**

The online version contains supplementary material available at 10.1186/s40001-022-00756-2.

## Background

Colorectal cancer (CRC) is one of the most common malignancies worldwide. It is the third most frequent leading cause of death of all the malignancies worldwide [1, 2]. CRC pathogenesis is complex and associated with several risk factors, including alcohol intake, obesity, cigarette smoking, consumption of processed and red meat, and inflammatory bowel disease [3, 4]. CRC may be sporadic or familial [5, 6]. Sporadic cases with no familial history comprise approximately 65% of CRC [7], with the remaining 35% of patients having an inherited form of the disease [8]. The progression of CRC is purportedly caused by chromosome instability and the stepwise accumulation of mutations in oncogenes and tumor suppressor genes [9, 10]. The inactivation of tumor suppression genes p53, retinoblastoma (Rb), adenomatous polyposis coli (APC), oncogenic RAS mutations, and over-expression of β-catenin are common in CRC progression [11, 12].

Infectious agents may contribute to the development of human cancers [13]. Human polyomavirus JC virus (JCPyV) was first identified as an etiologic agent in progressive multifocal encephalopathy (PML) [14]. JCPyV is a non-enveloped, double-stranded DNA virus [15], with three major regions defined in the viral genome: regulatory, early, and late regions [15, 16]. The regulatory region contains promoters for early and late transcripts, and a replication origin; the early region encodes for small t and large T antigens; and the late region encodes for structural proteins VP1, VP2, VP3, and an agnoprotein. The association of JCPyV with tumors has been established in experimental animals [17]. In transformed cells infected with JCPyV, an integrated JCPyV genome that expressed viral large tumor antigen (LT) also interacted with tumor suppressor genes p53 and Rb, resulting in uncontrolled cellular proliferation [18]. JCPyV has been detected in human cancers [19, 20], and its association with CRC has been reported by many studies [21–23]. The JCPyV DNA sequences are highly prevalent in the human upper and lower gastrointestinal tract of immunocompetent individuals [22, 24]. JCpyV LT can inactivate p53 and Rb, and enhance the nuclear localization of β-catenin, activating transcription of its downstream target genes [21]. A prototype strain, Mad-1, is the only strain detected in CRC, but not in non-neoplastic tissues [23]. Therefore, JCPyV is thought to induce chromosome instability in colon tissue, and promote colorectal tumorigenesis [25, 26]. Previously, we found a high prevalence of JCPyV in CRC, with the archetypal JCPyV, not the Mad-1, being the predominant genotype in Taiwan [27]. We hypothesized that JCPyV infection may contribute to CRC progression. The association of JCPyV with CRC progression is not conclusive and remains to be determined. Here, we analyzed the presence of JCPyV in different stages of CRC and sequenced the viral genotypes in CRC samples. The results showed that the progression of CRC led to an increase in the proportion of samples with JCPyV DNA, indicating that there may be an association between JCPyV and CRC progression.

## Methods

### Clinical specimens

We collected formalin-fixed paraffin-embedded (FFPE) tissue blocks from the Biobank of Ditmanson Medical Chiayi Christian Hospital from 2016 to 2018, where patients with CRC had undergone resection of primary tumors. The staging of tumors was evaluated at the time of diagnosis using Astler–Coller’s modification of the Dukes’ staging system [28], and the pathological classification of tumors was performed according to World Health Organization (WHO) [29]. The inclusion criteria were patients who were diagnosed with CRC, and tissue samples with more than 50% of tumour area. Patients with a history of other cancers, who were immunocompromised, or had post-transplant organs were excluded from this study. The clinical characteristics of the patients are provided in Additional file 1: Table S1. A total of 95 tissue blocks were included in this study: 20 tissue samples of stage I, and 25 samples each of stages II, III, and IV. The patients’ age ranged from 31 to 81 years. This study was performed in line with the principles of the Declaration of Helsinki. The Institutional Review Board at the Ditmanson Medical Chiayi Christian Hospital approved the study; written informed consent was obtained from all study subjects, and stored in the Biobank (Date: 2020/05/20; IRB approved No. 2020011, and Biobank approved No. OBD2020001).

### DNA extraction

Nucleic acid extraction from FFPE tissues was performed using the Qiagen^™^ DNeasy kit, according to the manufacturer’s instructions (Qiagen, Hilden, Germany). A total of 200 ng of DNA was used for the nested PCR, to detect viral DNA in the tissue samples.

### Nested PCR

The presence of JC polyomavirus was determined from 200 ng of extracted DNA by nested PCR, using two pairs of primers annealed to the regulatory region of JCPyV. The primer pairs JBR1 and JBR2 were used for the first PCR, and primer pairs JBRNS and JBRNAS were used for the nested PCR (Table 1). The PCR product was expected to be between 200 and 300 bp after nested PCR amplification [27, 30]. The process of formalin fixation and paraffin embedding may cause damage to nucleic acids in tissue samples [31]. We amplified the β-actin genes (135 bp) as an internal control, to ascertain the quality of FFPE CRC tissues [32]. The forward and reverse primer sequences for β-actin genes are as follows: 5ʹ-AGCGGGAAATCGTGCGTG-3ʹ, and 5ʹ-GGTGATGACCTGGCCGTC-3ʹ, respectively. The JCPyV (Accession No. U61771) and BKPyV (Accession No. DQ305492) genomic DNA were cloned into plasmids and used as a positive control. Accordingly, the nested PCR fragment of BKPyV is larger than that of JCPyV [16, 33]. The nested PCR products were analyzed by electrophoresis in a 2.5% agarose (molecular-biology grade; IBI Biotechnologies, New Haven, CT) gel. The gel was stained with ethidium bromide (Sigma-Aldrich, Inc., St. Louis, MO, USA), and imaged under UV light. The nested PCR products were then cleaned up by GeneMark DNA clean extraction kit (GeneMark, Taipei, Taiwan), and sequenced by Tri-I Biotech, Inc. (Taipei, Taiwan), to determine the DNA sequence.

**Table 1:** Primer sequences for nested PCR analysis

Primer name	Sequence (5'–3')	Nucleotides (Accession. No. U61771)
JBR1	F-CCTCCACGCCCTTACTACTTCTGAG	5067–5091
JBR2	R-GTGACAGCTGGCGAAGAACCATGGC	279–255
JBRNS	F-GAGGCGGCCTCGGCCTC	5100–5
JBRNAS	R-GGCTCGCAAAACATGT	227–212

## Results

**Fig. 1:**
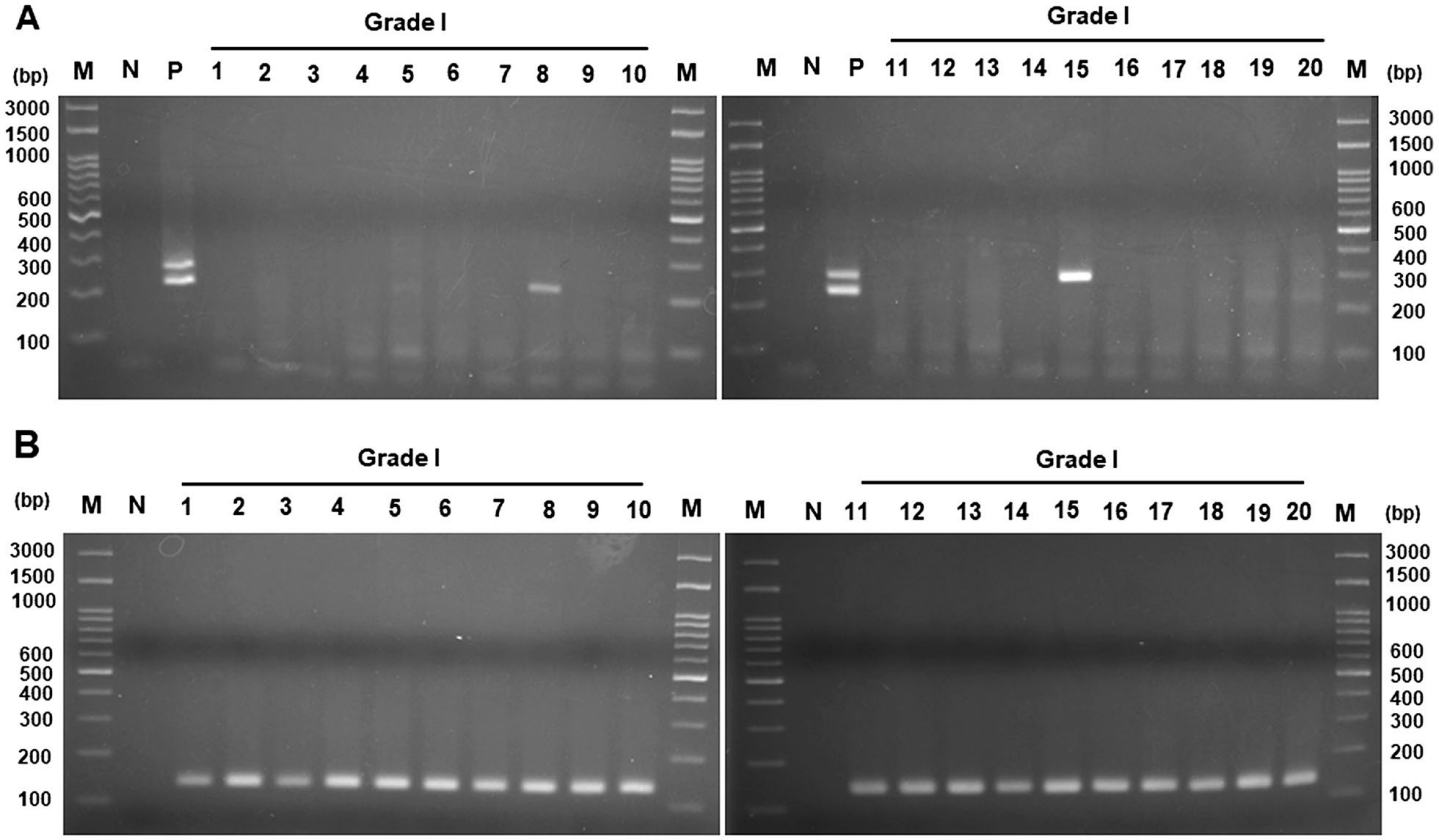
Electrophoresis of DNA fragments in stage I CRC samples. **A** Viral regulatory region was amplified by nested PCR, using JC-BK-specific primers. **B** Beta-actin gene was amplified by specific primers. The product was analyzed by electrophoresis in 2.5% agarose gel. Following electrophoresis, the gel was stained with ethidium bromide and photographed under UV light. JCPyV and BKPyV genomic DNA were cloned into a plasmid and used as a positive control. *M* marker, Lane numbers represent the number of the tissue sample involved. *N* negative control (without DNA)

To determine the presence of viral DNA in different stages of CRC tissues, we used two pairs of primers annealing to the constant region of both JCPyV and BKPyV, by nested PCR analysis [27, 30]. As Fig. 1 shows, two DNA fragments (case No. 8 and 15) between 200 and 300 bp were amplified out of 20 stage I colorectal cancer tissues (Fig. 1A). The size of the amplified DNA fragments was similar to that of the JCPyV positive control. The β-actin fragment could be amplified in all the analyzed stage I CRC tissues (Fig. 1B); this indicated the quality of DNA samples was acceptable for PCR amplification. Therefore, the ratio of positive DNA detection was 10% (2/20) in grade I CRC tissues.

**Fig. 2:**
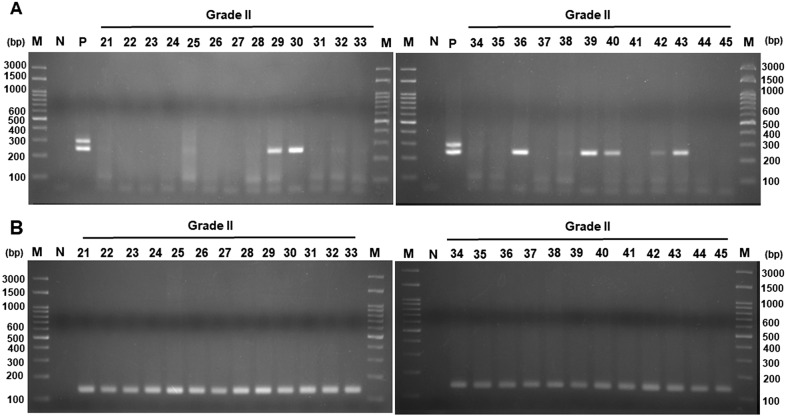
Electrophoresis of DNA fragments in stage II CRC samples. **A** Viral regulatory region was amplified by nested PCR, using JC-BK-specific primers. **B** Beta-actin gene was amplified by specific primers. The product was analyzed by electrophoresis in 2.5% agarose gel. Following electrophoresis, the gel was stained with ethidium bromide and photographed under UV light. JCPyV and BKPyV genomic DNA were cloned into a plasmid and used as a positive control. *M* marker, Lane numbers represent the number of the tissue sample involved. *N* negative control (without DNA)

We then detected the viral DNA in 25 stage II CRC tissues. After nested PCR amplification and analysis by agarose gel electrophoresis, seven DNA fragments (cases No. 29, 30, 36, 39, 40, 42, and 43) were amplified out of 25 stage II CRC tissues (Fig. 2A). Beta-actin DNA fragments could be detected in all the analyzed CRC tissues (Fig. 2B), making the ratio of positive DNA detection 28% (7/25) in grade II CRC tissues. The results in Figs. 1 and 2 indicate that the proportion of positive viral DNA increased with the progression of CRC stage.

**Fig. 3:**
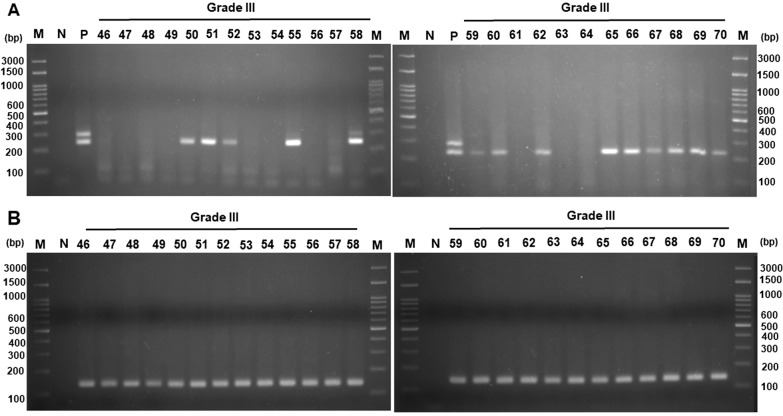
Electrophoresis of DNA fragments in stage III CRC samples. **A** Viral regulatory region was amplified by nested PCR, using JC-BK-specific primers. **B** Beta-actin gene was amplified by specific primers. The product was analyzed by electrophoresis in 2.5% agarose gel. Following electrophoresis, the gel was stained with ethidium bromide and photographed under UV light. JCPyV and BKPyV genomic DNA were cloned into a plasmid and used as a positive control. *M* marker, Lane numbers represent the number of the tissue sample involved. *N* negative control (without DNA)

**Fig. 4:**
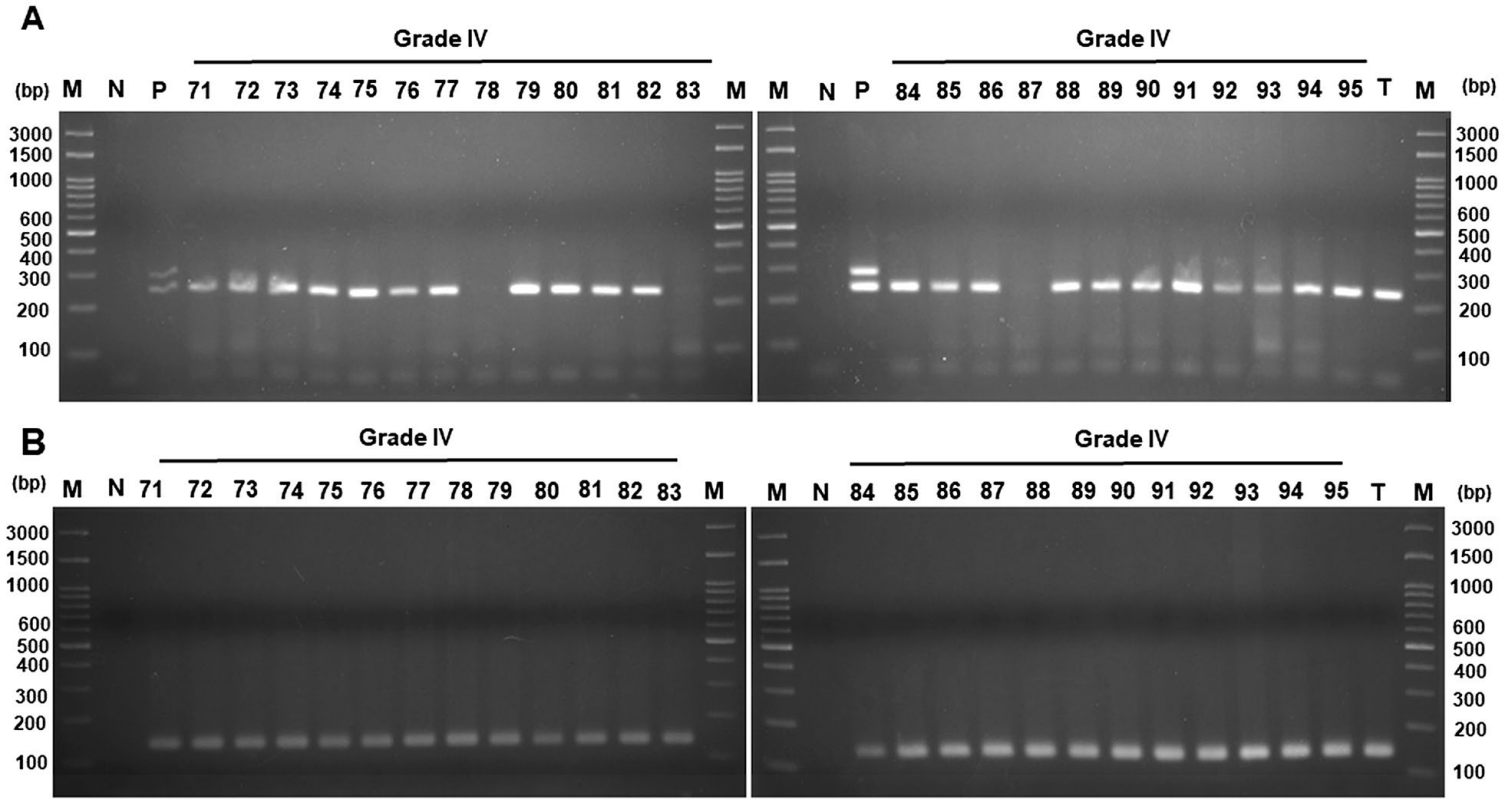
Electrophoresis of DNA fragments in stage IV CRC samples. **A** Viral regulatory region was amplified by nested PCR, using JC-BK-specific primers. **B** Beta-actin gene was amplified by specific primers. The product was analyzed by electrophoresis in 2.5% agarose gel. Following electrophoresis, the gel was stained with ethidium bromide and photographed under UV light. JCPyV and BKPyV genomic DNA were cloned into a plasmid and used as a positive control. *M* marker, Lane numbers represent the number of the tissue sample involved. *N* negative control (without DNA). T: a tested sample was run aligned with the CRC tissues

We then detected the viral DNA by nested PCR and confirmed the quality of FFPE stage III and IV CRC tissues by amplification of β-actin DNA. Figure 3 shows that 14 DNA fragments (cases No. 50, 51, 52, 55, 58, 59, 60, 62, 65, 66, 67, 68, 69, and 70) were amplified out of 25 stage III colorectal cancer tissues (Fig. 3A). Out of 25 stage IV colorectal cancer tissues, the presence of DNA fragments between 200 and 300 bp after nested PCR amplification in agarose gel electrophoresis (Fig. 4A) was shown in 22 (exceptions were cases No. 78, 83, and 87). Beta-actin DNA fragments could be detected in all the analyzed stage III and IV colorectal cancer tissues (Figs. 3B and 4B). The size of the amplified DNA fragments was similar to that of the JCPyV positive control.

These results demonstrated that the positive ratio of DNA was 10% (2/20), 28% (7/25), 56% (14/25), and 88% (22/25) in grades I, II, III, and IV CRC tissues, respectively. The positive ratio increased with the progression of CRC stages. We propose that the DNA fragments amplified from colorectal tissues are JCPyV, since the length of the DNA fragment is similar to that of JCPyV amplified by these two primer pairs of nested PCR.

**Fig. 5:**
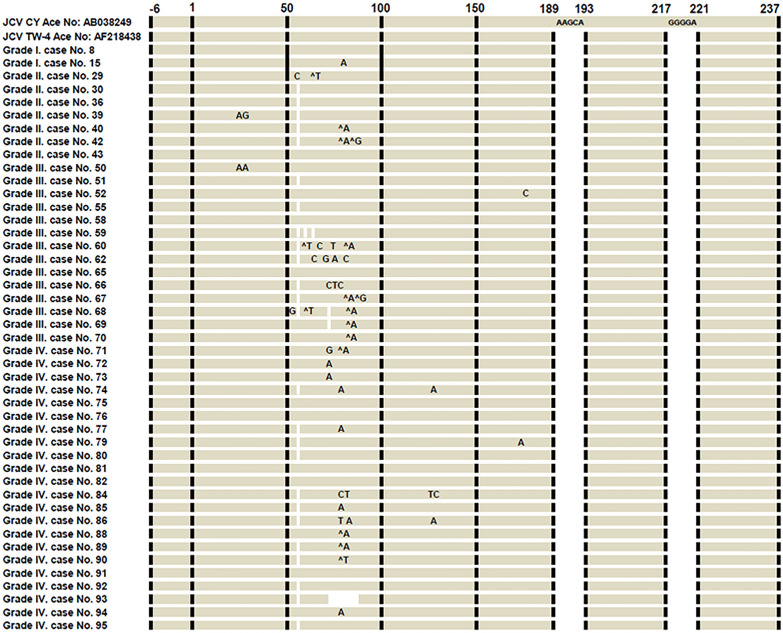
Schematic representation of the JCPyV regulatory region identified in the CRC tissues. Regulatory regions of CY and TW4 are shown for comparison. (ATCG): point mutation; (^) insertion; (□) deletion

All the positive samples were directly sequenced and blasted against the nucleotide database by Needleman–Wunsch Global Align Nucleotide software provided by NCBI blast tools, to confirm that the nested PCR products were viral DNA. As shown in Fig. 5, most sequences exhibit two deletions, one from nucleotide 189 to 193, and another from 217 to 221 of the archetype JCPyV (GeneBank accession no. AB038249). The detected sequence is similar to JCPyV TW-4 (GeneBank accession no. AF218438). Our study found no rearrangement in the amplified regulatory region, indicating archetypal-like strains of JCPyV were present in CRC tissues in Taiwan.

## Discussion

In the current study, we found that the proportion of tissues containing JCPyV DNA increased from 10%, 28%, 56%, and 88% alongside the progression of CRC stages. The archetypal-like JCPyV was the predominant genotype detected in CRC tissues. Some changes in viral sequences, such as mutations, insertions, or deletions, were found in these amplicons. The mutation rate in the viral sequence was not associated with CRC progression. We believe the findings support the hypothesis that JCPyV infection may be related to the progression of CRC.

It is thought that the LT protein of polyomavirus contributes to viral transformation activity [34, 35], and that β-catenin is stabilized by JCPyV LT protein [21], causing JCPyV to play a role in the progression of CRC [36]. Recently, increasing evidence suggests various pathogens represent risk factors in CRC oncogenesis [37, 38]. Among these infectious agents, Epstein–Barr virus (EBV), human papillomavirus (HPV), and JCPyV, have been intensively studied [38]. However, some controversial data have also been reported [39]. The inconsistency in these studies may be due to the different detection methods used in different countries. In the current study, we found that the proportion of the JCPyV genome increased as the stages of CRC samples progressed, suggesting that JCPyV might be a cofactor ino CRC progression.

Ricciardiello et al. reported that the rearrangement Mad-1 genotype was the only JCPyV strain amplified in CRC tissues [23]. Previously, we found that the archetypal JC polyomavirus was the primary genotype found in CRC samples in Taiwan [27]. This contradictory data may also be due to the difference between detection methods used in different countries. In the current study, we did not encounter a correlation between viral genotype variation with CRC progression, suggesting that the genotype is not the main factor contributing to CRC progression.

## Conclusion

Altogether, the results of our study appear to support the hypothesis that JCPyV may contribute to CRC progression.

## Supplementary Information

**Additional file 1: Table S1.** Characteristics of colorectal cancer samples and summary of the analysis of human polyomavirus DNA.

## Data Availability

The data sets generated and/or analyzed during the current study are available from the corresponding author on reasonable request.
