# Supplementary material for: Unusually high incidence of polyomavirus JC infection in the higher grade of colorectal cancer tissues in Taiwan
Source: Eur J Med Res. 2022 Jul 20;27:127. doi: 10.1186/s40001-022-00756-2 (PMC9301828; doi:10.1186/s40001-022-00756-2)
Supplement: Supplementary file 1 — Additional file 1: Table S1. Characteristics of colorectal cancer samples and summary of the analysis of human polyomavirus DNA. [file 40001_2022_756_MOESM1_ESM.docx]

| Table S1 | Characteristics of colorectal cancer samples and summary of the analysis of human polyomavirus DNA | | | | |
| --- | --- | --- | --- | --- | --- |
|  |  |  |  |  |  |
| CRS no. | Age (yrs)/Sex | TNM | stage | Detection methods | |
|  |  |  |  | Nested PCR | Sequence |
| 1 | 74/M | T1N0M0 | I | - | ND |
| 2 | 78/F | T2N0M0 | I | - | ND |
| 3 | 47/M | T2N0M0 | I | - | ND |
| 4 | 73/F | T2N0M0 | I | - | ND |
| 5 | 58/M | T2N0M0 | I | - | ND |
| 6 | 81/M | T2N0M0 | I | - | ND |
| 7 | 63/F | T1N0M0 | I | - | ND |
| 8 | 71/M | T2N0M0 | I | + | TW-4 like |
| 9 | 51/M | T2N0M0 | I | - | ND |
| 10 | 68/F | T1N0M0 | I | - | ND |
| 11 | 72/F | T2N0M0 | I | - | ND |
| 12 | 51/F | T1N0M0 | I | - | ND |
| 13 | 68/F | T2N0M0 | I | - | ND |
| 14 | 71/M | T2N0M0 | I | - | ND |
| 15 | 76/M | T2N0M0 | I | + | TW-4 like |
| 16 | 76/M | T2N0M0 | I | - | ND |
| 17 | 66M | T2N0M0 | I | - | ND |
| 18 | 76/M | T2N0M0 | I | - | ND |
| 19 | 64/M | T2N0M0 | I | - | ND |
| 20 | 58/F | T1N0M0 | I | - | ND |
| 21 | 74/M | T3N0M0 | IIA | - | ND |
| 22 | 57/F | T3N0M0 | IIA | - | ND |
| 23 | 80/F | T3N0M0 | IIA | - | ND |
| 24 | 60/M | T3N0M0 | IIA | - | ND |
| 25 | 59/F | T3N0M0 | IIA | - | ND |
| 26 | 71/M | T3N0M0 | IIA | - | ND |
| 27 | 62/M | T3N0M0 | IIA | - | ND |
| 28 | 68/M | T3N0M0 | IIA | - | ND |
| 29 | 74/M | T3N0M0 | IIA | + | TW-4 like |
| 30 | 69/M | T3N0M0 | IIA | + | TW-4 like |
| 31 | 68/M | T3N0M0 | IIA | - | ND |
| 32 | 68/F | T3N0M0 | IIA | - | ND |
| 33 | 70/M | T3N0M0 | IIA | - | ND |
| 34 | 61/F | T3N0M0 | IIA | - | ND |
| 35 | 49/M | T4aN0M0 | IIB | - | ND |
| 36 | 65/M | T3N0M0 | IIA | + | TW-4 like |
| 37 | 62/M | T4bN0M0 | IIC | - | ND |
| 38 | 57/M | T4aN0M0 | IIB | - | ND |
| 39 | 63/M | T3N0M0 | IIA | + | TW-4 like |
| 40 | 74/M | T3N0M0 | IIA | + | TW-4 like |
| 41 | 61/F | T3N0M0 | IIA | - | ND |
| 42 | 63/M | T3N0M0 | IIA | + | TW-4 like |
| 43 | 69/M | T3N0M0 | IIA | + | TW-4 like |
| 44 | 45/M | T3N0M0 | IIA | - | ND |
| 45 | 80/F | T3N0M0 | IIA | - | ND |
| 46 | 69/M | T2N1bN0 | IIIA | - | ND |
| 47 | 59/M | T3N1cM0 | IIIB | - | ND |
| 48 | 46/F | T3N2bM0 | IIIC | - | ND |
| 49 | 55/F | T3N2bM0 | IIIC | - | ND |
| 50 | 68/F | T3N1aM0 | IIIB | + | TW-4 like |
| 51 | 57/F | T3N2aM0 | IIIB | + | TW-4 like |
| 52 | 60/F | T3N1aM0 | IIIB | + | TW-4 like |
| 53 | 75/M | T3N2bM0 | IIIC | - | ND |
| 54 | 59/F | T4aN2bM0 | IIIC | - | ND |
| 55 | 67/M | T3N2aM0 | IIIB | + | TW-4 like |
| 56 | 74/F | T3N1bM0 | IIIB | - | ND |
| 57 | 57/M | T3N2bM0 | IIIC | - | ND |
| 58 | 69/M | T3N1bM0 | IIIB | + | TW-4 like |
| 59 | 34/F | T3N1aM0 | IIIB | + | TW-4 like |
| 60 | 55/F | T4aN2bM0 | IIIC | + | TW-4 like |
| 61 | 43/F | T3N2bM0 | IIIC | - | ND |
| 62 | 54/M | T3N1bM0 | IIIB | + | TW-4 like |
| 63 | 78/M | T3N2bM0 | IIIC | - | ND |
| 64 | 31/M | T3N2bM0 | IIIC | - | ND |
| 65 | 69/M | T3N1cM0 | IIIB | + | TW-4 like |
| 66 | 65/F | T3N1bM0 | IIIB | + | TW-4 like |
| 67 | 65/M | T3N1aM0 | IIIB | + | TW-4 like |
| 68 | 79/F | T3N1bM0 | IIIB | + | TW-4 like |
| 69 | 51/M | T3N1aM0 | IIIB | + | TW-4 like |
| 70 | 64/F | T3N2aM0 | IIIB | + | TW-4 like |
| 71 | 64/M | T3N2bN1a | IVA | + | TW-4 like |
| 72 | 65/M | T3N1bM1a | IVA | + | TW-4 like |
| 73 | 80/M | T4aN2bM1b | IVB | + | TW-4 like |
| 74 | 45/M | T4bN0M1b | IVB | + | TW-4 like |
| 75 | 62/F | T4bN0M1a | IVA | + | TW-4 like |
| 76 | 57/M | T4bN1aM1a | IVA | + | TW-4 like |
| 77 | 67/M | T4aN2bM1b | IVB | + | TW-4 like |
| 78 | 76/M | T4bN2bM1b | IVB | - | ND |
| 79 | 58/M | T2N1bM1a | IVA | + | TW-4 like |
| 80 | 62/M | T4bN2bM1a | IVA | + | TW-4 like |
| 81 | 73/F | T4bN2bM1b | IVB | + | TW-4 like |
| 82 | 63/M | T4aN2aM1b | IVB | + | TW-4 like |
| 83 | 54/F | T3N2bM1a | IVA | - | ND |
| 84 | 35/F | T3N2bM1a | IVA | + | TW-4 like |
| 85 | 72/M | T3N2aM1a | IVA | + | TW-4 like |
| 86 | 63/M | T3N1aM1a | IVA | + | TW-4 like |
| 87 | 55/M | T4aN2aM1b | IVB | - | ND |
| 88 | 65/M | T4aN0M1b | IVB | + | TW-4 like |
| 89 | 76/M | T4bN2aM1b | IVB | + | TW-4 like |
| 90 | 79/M | T3N2bM1a | IVA | + | TW-4 like |
| 91 | 45/M | T4aN2bM1b | IVB | + | TW-4 like |
| 92 | 50/F | T3N2bM1b | IVB | + | TW-4 like |
| 93 | 69/M | T3N1aM1a | IVA | + | TW-4 like |
| 94 | 66/M | T3N1cM1a | IVA | + | TW-4 like |
| 95 | 55/M | T3N1bM1 | IV | + | TW-4 like |
|  |  |  |  |  |  |

M: male; F: female. TNM: colorectal cancer staging was determined by the American Joint Committee on Cancer (AJCC)  TNM classification of malignant z; tumors. "+": positive. "-": negative. ND: not detected. TW4 (GeneBank accession no. AF218438).
